# Unraveling neuroprotection with Kv1.3 potassium channel blockade by a scorpion venom peptide

**DOI:** 10.1038/s41598-024-79152-1

**Published:** 2024-11-13

**Authors:** Emidio Beraldo-Neto, Vanessa Florentino Ferreira, Hugo Vigerelli, Karolina Rosa Fernandes, Maria Aparecida Juliano, Ana Leonor Abrahao Nencioni, Daniel Carvalho Pimenta

**Affiliations:** 1 Biochemistry Laboratory, Butantan Institute, São Paulo, Brazil; 2 Pharmacology Laboratory, Butantan Institute, São Paulo, Brazil; 3 Genetics Laboratory, Butantan Institute, São Paulo, Brazil; 4 Department of Biophysics, Escola Paulista de Medicina, Universidade Federal de São Paulo, São Paulo, SP Brazil

**Keywords:** Voltage-gated potassium channel, Neurotoxin, Hippocampus, Scorpion toxin, Kv1.3, αKtx12, Peptide, Biochemistry, Biological techniques, Cell biology, Drug discovery, Neuroscience

## Abstract

Voltage-gated potassium channels play a crucial role in cellular repolarization and are potential therapeutic targets in neuroinflammatory disorders and neurodegenerative diseases. This study explores *Tityus bahiensis* scorpion venom for neuroactive peptides. We identified the αKtx12 peptide as a potent neuroprotective agent. In SH-SY5Y cells, αKtx12 significantly enhances viability, validating its pharmacological potential. And in the animal model, we elucidate central nervous system (CNS) mechanism of αKtx12 through neuroproteomic analyses highlighting αKtx12 as a valuable tool for characterizing neuroplasticity and neurotropism, revealing its ability to elicit more physiological responses. The peptide’s potential to promote cell proliferation and neuroprotection suggests a role in functional recovery from nervous system injury or disease. This research unveils the neuroactive potential of scorpion venom-derived αKtx12’s, offering insights into its pharmacological utility. The peptide’s impact on neuronal processes suggests a promising avenue for therapeutic development, particularly in neurodegenerative conditions.

**Supplementary Information:**

The online version contains supplementary material available at 10.1038/s41598-024-79152-1.

## Introduction

Voltage-gated potassium (Kv) channels constitute a diverse family of transmembrane proteins that facilitate the outward movement of potassium ions (K^+^) within biological systems^1^. These channels are categorized into three distinct groups based on their predicted membrane topology, featuring six, four, or two transmembrane domains^2^. However, irrespective of their domain configuration, the subunits collectively form a single transmembrane pore for K^+^ efflux^3^. Kv channels are acutely responsive to alterations in the cellular membrane potential, and are predominantly implicated in cell repolarization processes^3^.

Several subtypes of these channels have emerged as potential targets for therapeutic intervention. For instance, the Kv1.3 channel has garnered attention as a promising therapeutic target in the context of neuroinflammatory disorders, due to its pivotal role in specific microglial cells^4^.

The microglia play a crucial role in the immune response of the central nervous system, and their activation has been implicated in the pathogenesis of neurodegenerative diseases. Studies show that the expression of Kv1.3 is significantly increased in microglia, suggesting that these channels may be involved in regulating the inflammatory response in the brain^5^. Its expression is also significantly increased in Alzheimer Disease^6^. Selective inhibition of Kv1.3 has been shown to reduce microglial activation and the production of pro-inflammatory cytokines, such as IL-1β and TNF-α, promoting a neuroprotective environment^7,8^. This modulation not only attenuates neuroinflammation but also alters the microglial phenotype to a less reactive state that is more conducive to neuronal survival. In neurons, studies have shown that modulation of Kv1.3 can directly impact neuronal function, with channel inhibition associated with preservation of synaptic plasticity and improved cognitive function^5,9^.

Peptides from scorpion venom are recognized for their interactions with ion channels^10^. The α-KTx family of scorpion toxins, is a potent inhibitor of Kv1.3, Kv1.2, and Shaker-type potassium channels^11^. This selective inhibition leads to reduced membrane hyperpolarization in microglia, which in turn diminishes their activation and the subsequent inflammatory response^12^. These characteristics make this family of toxins a promising candidate for modulating immune responses in the CNS, potentially reducing neuroinflammation and providing neuroprotection^13^.

Therefore, the primary objective of our study was to identify a peptide with neuroactive potential for therapeutic application in central nervous system (CNS) models. To achieve this, we employed a systematic approach involving the screening of *Tityus bahiensis* (Tb) scorpion venom, evaluating the effects of its chromatographic fractions on the viability of SH-SY5Y cells, a widely used neuroblastoma cell line model for neurodegenerative diseases. The promising results from these initial experiments led us to focus on a specific peptide, αKTx12, known for its inhibitory activity on Kv1.3 channels. This peptide demonstrated significant neuroprotective effects, validating its potential as a pharmacological tool for CNS applications. Following these findings, we advanced to animal model studies to further investigate the peptide’s mechanism of action, employing neuroproteomics to elucidate its role within the CNS.

## Results

The investigation of the neuroprotective biological activities of the Tb toxins started with the biomonitored venom fractionation and culminated in the selection of αKtx12, as shown in Supplementary Figures S1 and S2.

Figure S1, panel A, shows a representative chromatographic separation profile of the crude Tb venom, into 5 fractions (F1-5), selected according to the overall peak distribution throughout the chromatogram. This step was designed to group the major toxins, not to separate peptides.

Tb venom and its fractions were tested on the SH-SY5Y cell line to assess their cellular viability effects. Neither the venom nor the fractions exhibited cytotoxic effects at any of the concentrations tested. Notably, F3 and F4 demonstrated an increase in the cellular viability by 12,5 and 15% respectively (F = 9,55 *p* < 0.5), suggesting a potential cytoprotective or stimulatory effect (Supplementary Figure S1, panel B).

Due to the lower molecular diversity observed in F3, it was selected as the active fraction to be subjected to a secondary fractionation process, resulting in a series of chromatogram peaks, that were collected for further biological testing. The subfractions were sequentially labeled F3.1-3.5, and the relative concentration was 33%, 11%, 4.8%, 43%, and 7%, respectively (Supplementary Figure S2, panel A). These subfractions were subjected to further MTT viability assay the on SH-SY5Y cell line (Supplementary Figure S2, panel B). The subfraction F3.4 consistently replicated the results obtained with the initial fraction F3. It is worth noting that these analyses were primarily qualitative in nature, aimed at identifying the molecule responsible for the observed activity.

**Fig. 1:**
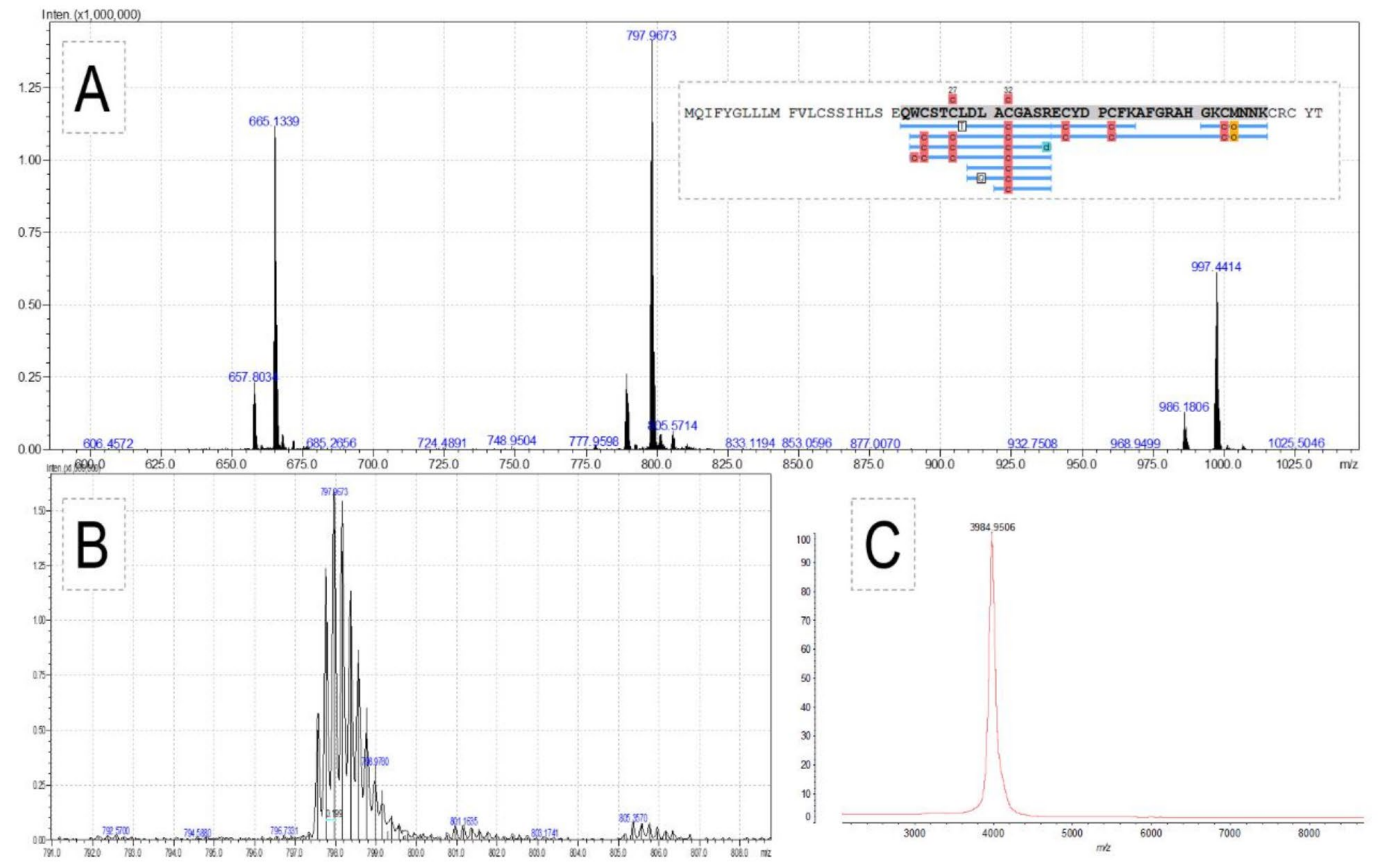
(**A**) Proteomic identification of α-Ktx 12 (UNIPROT A0A0C9S3A0 or P59936) was achieved through mass spectrometry, analyzed against a T.bahiensis transcriptomic database. (**B**) Spectrometric profile obtained through ESI-IT-TOF analysis, highlighting the 5 + charged ion with m/z 3985.14 Da (C**C** Spectrometric profile obtained through MALDI-TOF/MS analysis. A.

The subfraction F3.4 (134 ± 14), which exhibited a statistically significant (t = -4,11 *p* < 0.001) in relation to control group (102 ± 2) improvement in the cellular viability of SH-SY5Y cells (Figure S2, panel B), was characterized by mass spectrometry analyses (Fig. 1). This investigation, including the proteomic analyses, identified a major peptide of 3985.14 ± 0.55 Da, represented by the [M + 4 H]^4+^, [M + 5 H]^5+^, and [M + 6 H]^6+^ ions, and a minor peptide of 3940.8 Da (Fig. 1, panel A). In Fig. 1A the sequence of the proteomically identified αKtx12 trypsin-digested peptide, highlighted in blue, shows amino acid substitutions identified by Peaks Studio at residues 28 and 30, L◊T and L◊Q, respectively. The proteomic identification was performed using a transcriptomic sequence database (UniProt A0A0C9S3A0) deposited in the UNIPROT. Peaks Studio raw data processing identified F3.4 as αKtx12 toxin (UniProt P59936), with a logP value of 194.1. Figure 1, panel B shows the zoomed mass spectrometric profile of the [M + 5 H]^5+^ ion. Figure 1, panel C shows the MALDI-TOF/MS profile of the purified peptide. Two different mass spectrometric techniques and instruments were used in order to assess purity and molecular mass. The MALDI-TOF/MS analysis (Fig. 1, panel C) shows a single 3984.9506 m/z ion corresponding to the singly charged peptide, within the expected mass error for this technique.

In order to understand the possible mechanism of action of this peptide, animal models were used to evaluate neurophysiological pattern using histological and neuroproteomic techniques.

**Fig. 2:**
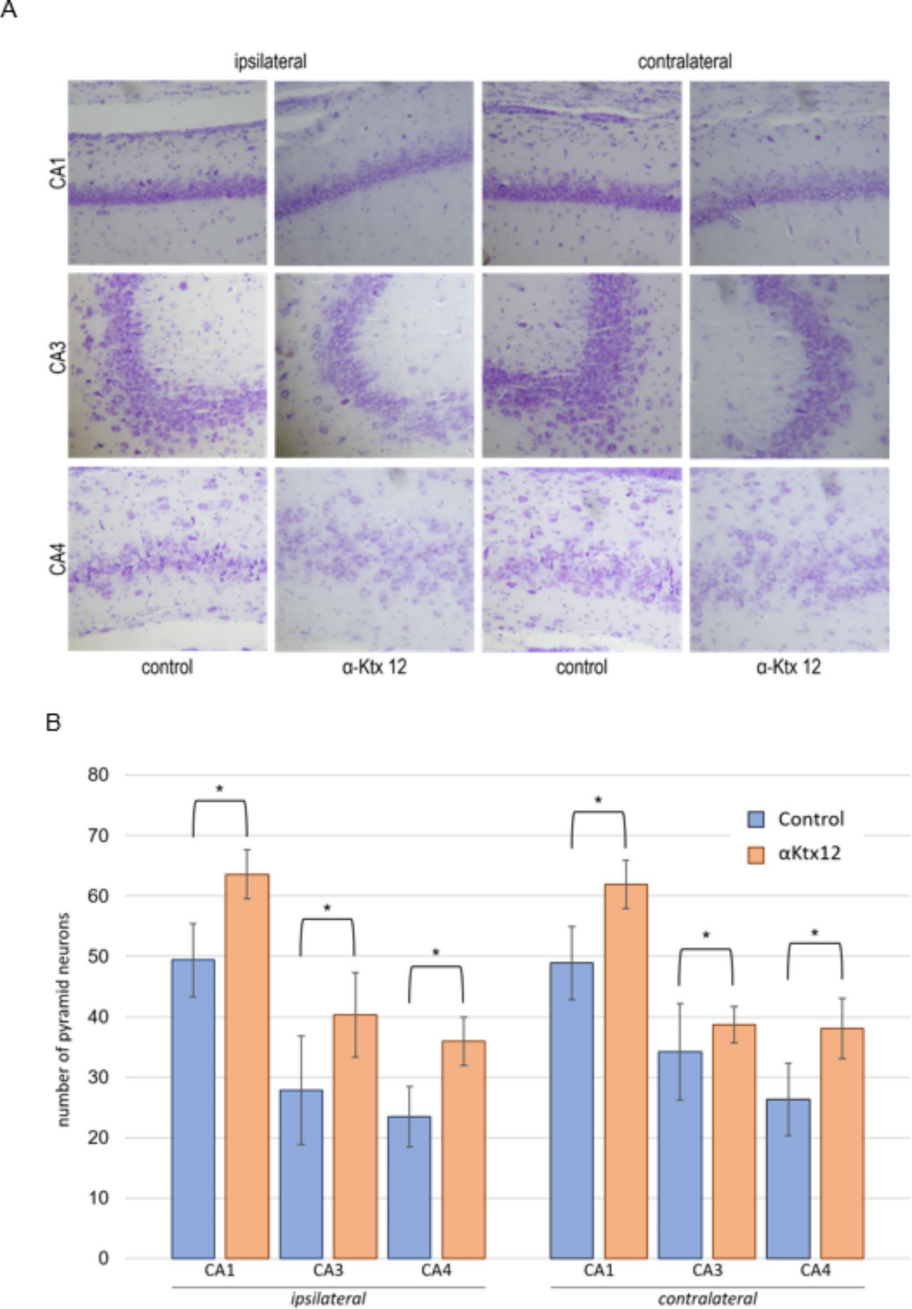
(**A**) Histological sections of the CA1, CA3, and CA4 regions of rat hippocampus. Ipsilateral and contralateral hippocampus of rats (*n* = 6) injected with α-Ktx 12 peptide. Analyzed by optical microscopy (5 μm) with a 40x objective lens. Stained with cresyl violet. (**B**) Statistical analysis of the number of pyramidal cells in histological sections of rat brain. Analysis of histological brain sections (5 μm) examined by optical microscopy with a 40x objective lens. The control group was injected with Ringer’s solution (2 µl) intrahippocampally, and the experimental group (*n* = 6) was injected with α-Ktx 12 peptide (2 µg/µl) intrahippocampally. The analysis pertains to the pyramidal layer in the hippocampal areas CA1, CA3, and CA4 on the treated (ipsilateral) and non-treated (contralateral) sides. Data is presented as means ± standard deviation. t-test. *indicates statistical significance against the respective control group (*p* < 0.05).

Histological analysis showed that αKtx12 injection increased the number of pyramidal cells in all regions of the hippocampus, both on the injection side (ipsilateral) and the contralateral side, compared to the Ringer’s solution (negative control) in an average of 35% increase across all regions of the hippocampus (Fig. 2, panels A and B).

The initial neuroproteomic analyses analysis employed was the Venn diagram, illustrating the number of identified proteins for each individual group as well as the shared/common proteins among them, as shown in supplementary Figure S3, which shows that the total number of detected proteins was similar among the groups: αKTx12: 610 proteins, 185 of which were unique; LPS: 594 proteins, 182 unique; and Ringer’s solution: 640 proteins and 211 unique. The number of shared proteins between the three conditions was about half of the total proteins for each group (282), whereas the shared proteins between two experimental conditions were around 10–15%, depending on the pair compared.

**Fig. 3:**
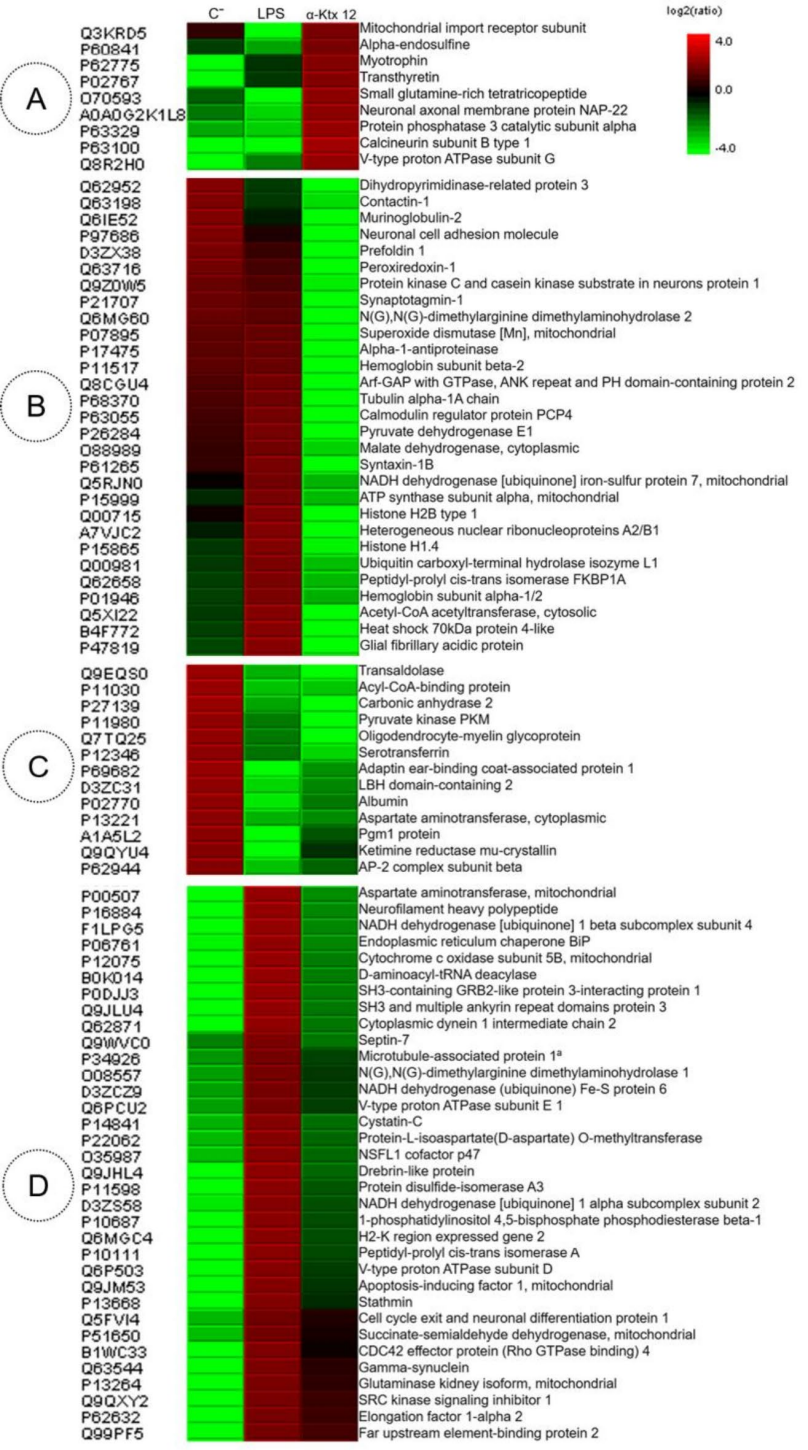
Label-free heatmap analysis of hippocampal tissue from the animals (*n* = 4), respectively from left to right: negative control group (Ringer’s solution), positive control group (LPS), and experimental group (α-Ktx 12). The log2(ratio) scale represents proteins, with higher concentrations shown in red and lower concentrations in green. UNIPROT codes and descriptive names are provided for protein identification. (**A**) Increased concentrations in the experimental group compared to LPS and negative control. (**B**) Decreased concentrations in the experimental group compared to the negative control and LPS. (**C**) Intermediate concentrations in the experimental group compared to LPS and decreased compared to the negative control. (**D**) Increased concentrations in the experimental group compared to the negative control and decreased compared to LPS.

Label-free proteomics analyses were also performed, selecting proteins with the most significant changes in the experimental group (Fig. 3). Each row represents a specific protein identified by the UNIPROT code and protein name. The three columns denote different experimental conditions: Ringer’s solution (control, C), LPS, and αKtx12. The color intensity ranges from green (low relative expression) to red (high relative expression). We have selected 85 proteins that were categorized into four groups selected based on the difference between the experimental group (αKTx12) and the two control groups (LPS and Ringer’s solution).

Figure 3, Subset A, contain 9 proteins, there were only relatively increased in in the experimental group, compared to both LPS and Ringer’s solution groups. Subset B, on the other hand, consists of 29 proteins that were primarily relatively decreased only in the experimental group compared to Ringer’s solution and LPS. Subset C, with 13 proteins, contains proteins that were decreased in the LPS and αKtx12 group, compared to Ringer’s solution. Finally, Subset D contains 34 proteins that were majorly increased in the LPS group, compared to Ringer’s solution and αKtx12 groups. Such variations will be discussed below.

**Fig. 4:**
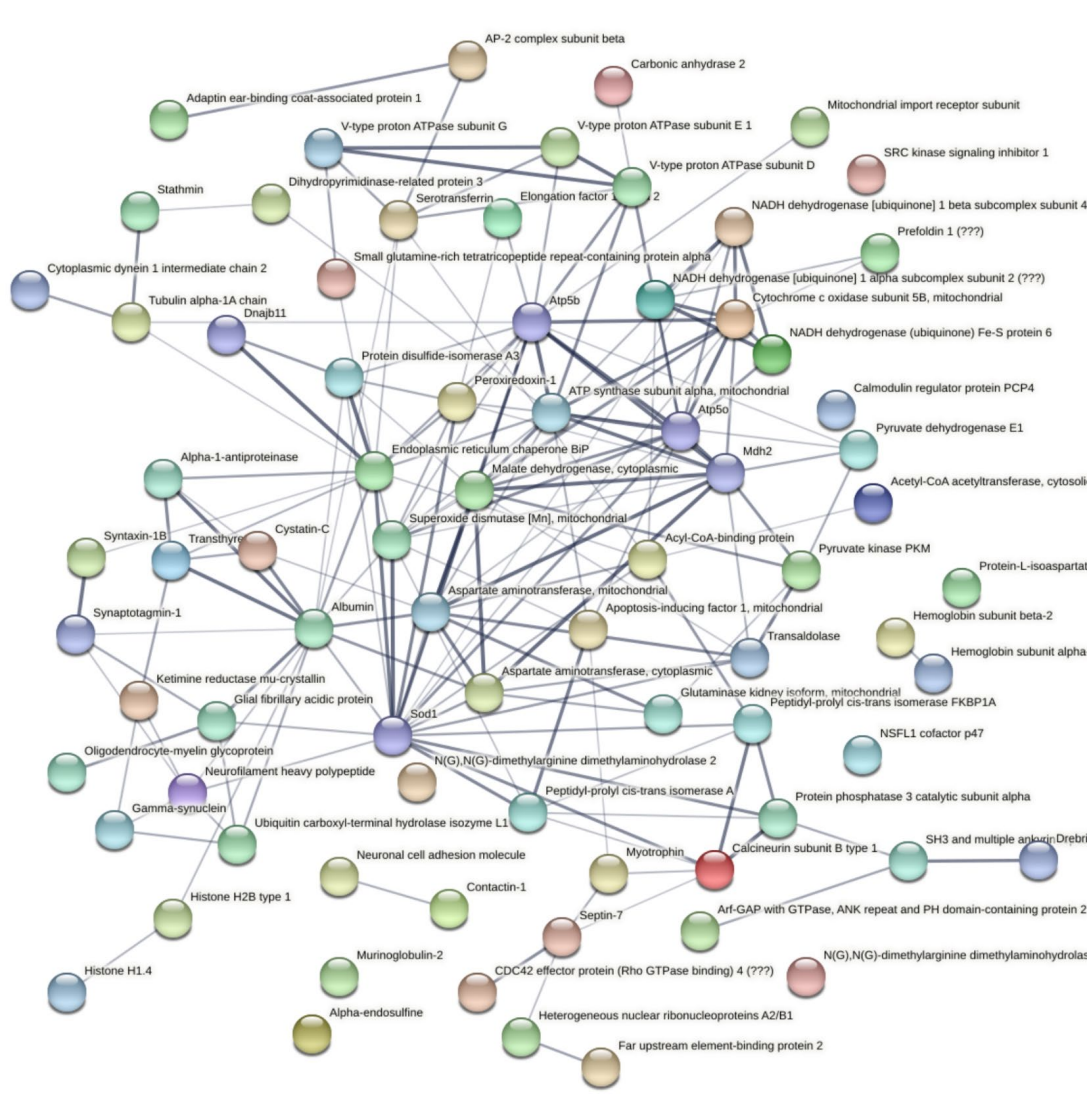
Functional protein interaction analysis using the STRING (Version 11.5) software for proteins identified through neuroproteomics conducted with Nano-LC-ESI-MS/MS equipment. Analysis was performed to unveil functional interactions among the dysregulated proteins identified from the hippocampi of the animals (*n* = 4).

In addition, we conducted a pathway analysis using STRING (V11.5), a tool designed to reveal functional interactions between proteins^14^. The proteins identified in the experimental group that exhibited alterations in the label-free proteomic analysis were subjected to this algorithm, and the results are depicted in Fig. 4. The analysis highlights potential interactions between proteins, represented by connecting lines, where the thickness of the line connecting the proteins indicates the confidence level of the association according to previously published supporting data, including experimental evidences such as co-expression, neighborhood, gene fusion and co-occurrence.

**Fig. 5:**
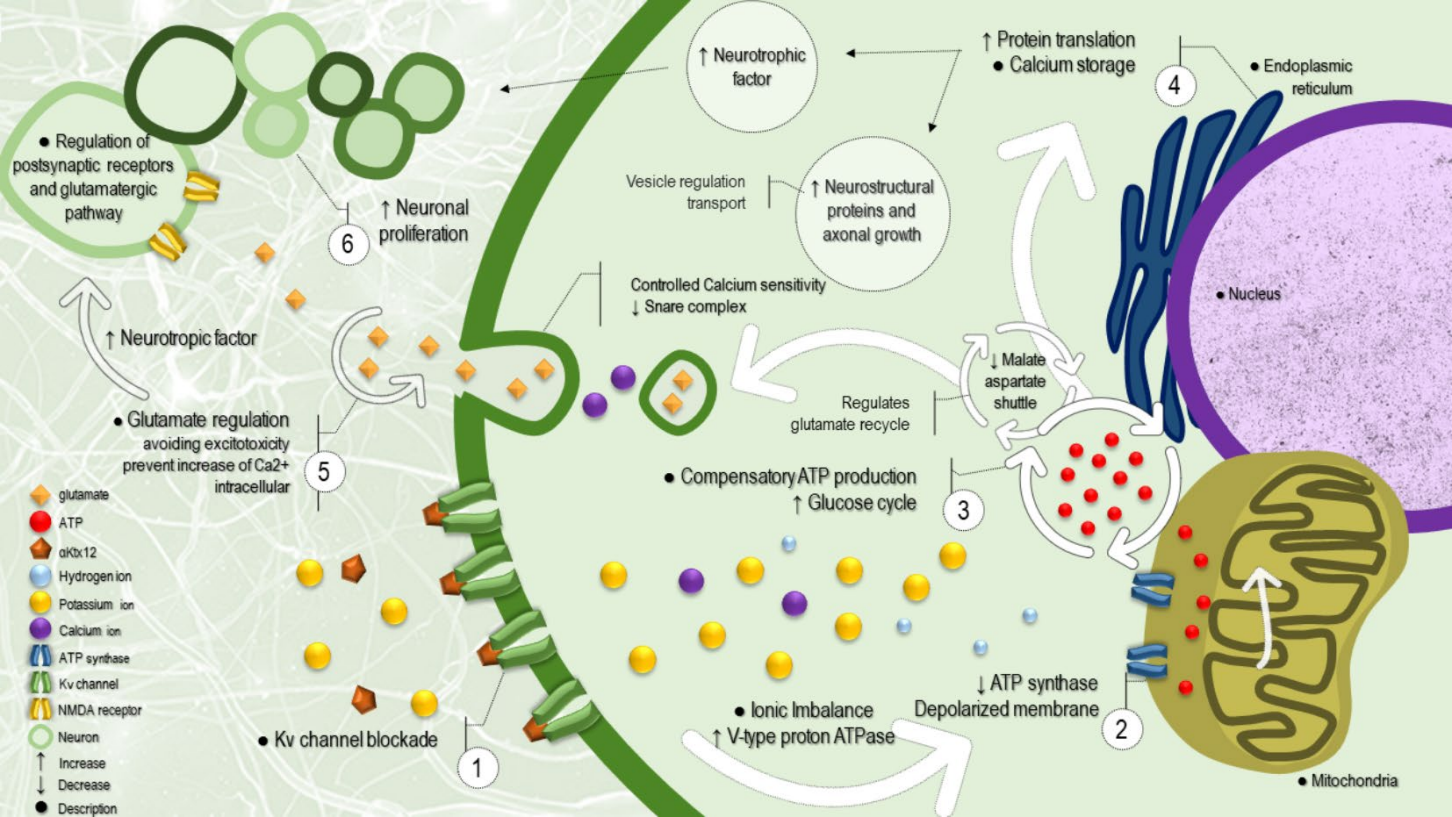
Proposed mechanism of action of the α-Ktx12 peptide in neurons: (1) Kv1.3 potassium channel blockade, (2) resulting ionic imbalance, (3) shifts in energy pathways towards glycolysis and regulation of glutamate metabolic pathways, (4) increased protein translation, including elevated neurotrophic factors and axonal growth proteins, (5) effects of regulated glutamate pathways preventing glutamatergic excitotoxicity, and (6) enhanced cell proliferation and/or neuroprotection.

This analytical approach served as a pivotal parameter in elucidating potential pathways and establishing a possible mechanism of action for the αKtx12 peptide, as shown in Fig. 5 and discussed below.

## Discussion

The possibilities of developing biotechnological tools derived from natural molecules, such as venoms, have already been proven to be efficient for different applications, including the pharmaceutical industry and applied scientific areas^15^.

In this work, we have screened the fractionated *T. bahiensis* venom for novel biological activities, particularly, we have evaluated the cell viability in SH-SY5Y cell lines after the treatment with the scorpion toxins. By performing biomonitored assays, we successfully isolated and proteomically identify αKtx12 peptide (Fig. 1) (UniProt code A0A0C9S3A0 or P59936) as the molecule responsible for the observed increase in the cell viability. Furthermore, this molecule acted as a potential neuroprotector and this particular effect was scrutinized by neuroproteomics and histological analyses, in animal models.

The αKtx12 peptide identified in this venom has two residue modifications (7 L◊T and 9 L◊Q) (Fig. 1) detected by the Peaks Studio software. This toxin has already been described as a potassium channel blocker, specifically targeting Kv1.3, Kv1.2, and Shaker subtypes^16^ The Kv1.3 channel has been considered as a therapeutic target for the treatment of neuroinflammatory disorders^17^. Kvs, in general, play a crucial role in neuronal regeneration and remyelination, essential factors for therapeutic strategies and recovery from demyelinating diseases and spinal cord injury^18^.

Therefore, aiming to evaluate the effects of this toxin on the neurophysiology of the animals tested, histological and neuroproteomic techniques were performed in this work. Stereotaxic surgery was performed on rats for the implantation of cannulas; subsequent intrahippocampal administration of the peptide was performed. One week after the peptide treatment, histological analyses of the brain tissue were performed and it was possible to evaluate and quantify an increase in the number of pyramidal cells from the CA1, CA3 and CA4 regions of the hippocampus in the αKtx12 treated group, compared to the control group (Fig. 2). This model was employed because pyramidal neurons in the hippocampus represent the most populous members of the excitatory family in cerebral regions. They represent approximately two-thirds of all neurons in the mammalian cerebral cortex and play a central role in numerous crucial cognitive processes^19^, making them a suitable neuronal model for study.

The assessment of neuroproteomic alterations in surgically excised tissue has proven to be intriguing due to its rich protein content. We performed Venn diagram analysis to verify the amounts of different proteins for each studied group (Suppl. Fig. S3). It is possible to observe that the total number of proteins detected in either the peptide-treated hippocampus or the control tissue did not differ significantly (610 vs. 640, respectively). However, label-free analysis (Fig. 3) shows that the relative quantity of the common proteins did vary between these two groups. Based on the label-free analyses and the string protein-network, the major metabolic pathways and the differentially expressed proteins are discussed below.

### Energy pathways

The energy pathway could be the beginning of the whole cascade of this metabolic shift that increased the number of neurons. An ionic imbalance event - consequence of the K^+^ deficiency – would upregulate proteins associated to ionic balance, such as V-type proton ATPase protein, which regulates ions^20^ and is increased in the experimental group (Fig. 3A). Our data also show that ATP synthase was decreased the experimental group (Fig. 3B). Typically, ATP synthase functions to generate ATP only when the mitochondria are sufficiently polarized^21^, that is, under normal physiological conditions and ionic balance. Under the experimental conditions, the lack of repolarization - due to the Kv channel blockade - would affect this state. Therefore, it would not be possible for the ATP synthase to generate ATP under the condition of loss of the electron transport chain. Ultimately, ATP hydrolysis would occur and protons would be pumped out of the mitochondrial^21^.

This situation may occur due to Kv channel blockade and the continuous lack of K^+^ ions (Figs. 5 − 1). This ATP deficiency, resulting from the ATP synthase mechanism (Figs. 5 − 2)., is one of the situations that could generate a feedback of energy compensation through other metabolic pathways that are more available during the selected time course of the experiments performed in the current work.

Table 1 shows proteins that were not identified in the control group, but only in the αKtx12 treated animals. It is possible to observe proteins such as fructose-bisphosphate aldolase and glycogen phosphorylase, which are directly involved in the glycolysis pathway^22^. The presence of these proteins indicates increased activity on the glycolytic pathway, with consequent increased ATP production, suggesting a potential mechanism for energy compensation due to the upregulation of this pathway (Figs. 5 − 3).

**Table 1:** Proteins exclusively identified (-10lgP > 50) in the α-Ktx 12 group in the proteomic analysis using Nano-LC-ESI-MS/MS equipment.

UNIPROT	-10lgP	Description
Q5XI72	196.71	Eukaryotic translation initiation factor 4 H
Q7TMB7	165.7	Phospholipid phosphatase-related protein type 4
D3ZTK0	137.49	Tetratricopeptide repeat domain 9B
P63100	136.73	Calcineurin subunit B type 1
P49806	93.52	Regulator of G-protein signaling 10
Q9JLT5	92.22	WFS1
P00884	77.49	Fructose-bisphosphate aldolase B
M0R513	73.51	Collagen type XXV alpha 1 chain
Q4KLG9	70.8	AN1-type zinc finger protein 2B
P18266	57.77	Glycogen synthase kinase-3 beta
F1MAE8	56.08	Growth differentiation factor 7

It is important to emphasize the need for ATP in the synaptic transmission, as this event requires large amounts of energy involved in the release and recycling of vesicles, in addition to (ATP) being essential for the generation of action potentials^23^. Thus, this is another factor that contributes to the increase in ATP, through the same event – the lack of repolarization due to the blockade of potassium channel.

This mechanism of switching energy pathways could be involved in the physiological regulation of neurotrophy (Figs. 5 − 4) and the likely resulting decrease in synaptic activity for adaptation of the neuronal system.

### Malate-aspartate shuttle

We highlight the malate-aspartate shuttle, in which malate dehydrogenase and aspartate aminotransferase primarily participate and were found to be relatively reduced in the αKtx12 group, compared to the control (Fig. 3B and D). According to the literature, these proteins are associated with both the citric acid cycle and the glutamate recycling metabolism^24^. Glutamate is an important excitatory neurotransmitter in the central nervous system. Its excessive release and action on glutamatergic ionotropic receptors induce an increase in intracellular calcium concentration, resulting in the activation of pro-apoptotic pathways^25,26^. The phenomenon called excitotoxicity is associated with chronic neurodegenerative disorders such as amyotrophic lateral sclerosis, multiple sclerosis, and Parkinson’s disease^27^. Commonly, studies of scorpion venoms peptides analyze this excitotoxicity pathway in models to analyze neuronal mechanisms^28,29^. However, the downregulation of these enzymes as a consequence of αKtx12 treatment (Fig. 5–5) may be directly related to the control of excitotoxicity, serving as one of the factors in the neuroprotective pathway, recycling and/or limiting the excessive production of glutamate to aspartate, thus preventing injury.

### Neuronal plasticity mechanisms

In neurons, protein translation for axon branching is dependent on actin^23^ - which, in turn, is regulated by Myotrophin^30^, (Fig. 3A) which was increased in the experimental group. Such protein translation may be stimulated by the energetic increase occurring in the axons and proteins such as eukaryotic translation initiation factor^31^ (Table 1) - found exclusively in the experimental group - and neuronal differentiation factor^32^ (Table 1; Fig. 3A) which, respectively, stimulate the translation initiation and consequently influence cell growth, proliferation, differentiation (Figs. 5–6). This relative protein pattern alteration, as detected by mass spectrometry, is ultimately reflected in the actual increase in the number of pyramidal neurons, as show in Fig. 2.

Among the neurotrophic factors, in addition to those already mentioned, we have also detected the growth differentiation factor 7 (Table 1). These factors act on the maintenance of the normal physiological function of the nervous system^33^, in cell survival^34,35^ and induce neurites outgrowth^36^. Their action is reported to be a consequence of their ability to interact and transduce cellular responses through Ras, which is, therefore, essential for the physiological function and plasticity of the nervous system^37^, and triggers distinct signal transduction pathways, such as the protein kinase cascade^38,39^ (Table 1; Fig. 3D). Therefore, the finding of these proteins on the exclusive or increased categories for the experimental group that corroborates the neurotropism observed in the biological experiments, as depicted in Fig. 2. The translation of mRNA promoting branching occurs only at axonal sites^23^ and the site of interaction of αKtx12 - the Kv1.3 channel – is, also, the axon^40^.

### Synaptic vesicles and Calcium Control

During action potential propagation, the exocytosis of the synaptic vesicle and the subsequent release of neurotransmitters at the presynaptic terminal occur within milliseconds^41,42^ and involve a number of proteins, some of which we found to be altered in the present study. Given the overall perspective of the pathways and in synergy with other identified mechanisms, we believe that this is a moment of reduced neuronal activity.

Proteins such as syntaxin and synaptotagmin (Fig. 3B), among others, are essential for the formation of the SNARE complex – which mediates the vesicle and membrane fusion during exocytosis^43^. Both proteins are decreased in the experimental group. Synaptotagmin, in particular, is responsible for triggering this fusion, mediating the coupling of an action potential for neurotransmitter release. Thus, not only the process, but also the entire complex is dependent on Ca^2+^, whose concentration is ultimately regulated by proteins^44^, such as calcineurin (Table 1) and calmodulin (Fig. 3B), which are increased and decreased, respectively, in the αKtx12 group. These proteins are biological sensors of Ca^2+^, and studies reveal that calcium entry pathways into the neuron differentially affect processes of development and neuronal plasticity, including cell survival, synaptic modulation, and calcium-mediated apoptosis^45^, according to cited literature.

These proteins act synchronously to maintain the physiological balance of synaptic vesicles^46^, and regulate their contents, stimulating the release of neurotransmitters and possible neurotrophic factors^47^, through the vesicles.

To synthesize these findings, we propose Fig. 5. This schema illustrates the proposed mechanism of action of αKtx12 in neurons, derived from the interpretation of the phenomena presented in the current study, combined with the literature reports. αKtx12 (Figs. 5 − 1) would bind to the potassium channel Kv1.3, blocking the efflux of potassium ions (K+) and inducing an ionic imbalance. This ionic imbalance results in a dysregulation of hydrogen ion (H^+^) concentrations (Figs. 5 − 2), leading to intracellular pH alteration and potential changes in enzymatic activity. Consequently, the intracellular pH alteration affects neuronal energy pathways, favoring glycolysis over oxidative phosphorylation (Figs. 5 − 3). This shift means that neurons produce more ATP from glucose breakdown while also generating more lactate as a byproduct. In addition, αKtx12 affects the regulation of glutamate metabolic pathways, a key excitatory neurotransmitter, potentially impacting synaptic transmission and neuronal plasticity.

Furthermore, increased glycolysis and lactate stimulate protein translation in neurons, resulting in increased expression of neurotrophic factors and axonal growth proteins (Figs. 5 − 4). These proteins play crucial roles in neuronal survival, development, and regeneration, particularly under conditions of injury or stress. Regulation of the glutamatergic pathway (Fig. 5–5) by αKtx12 also provides protection against excitotoxicity, a phenomenon that occurs when excessive glutamate enters the synaptic cleft, leading to an influx of calcium ions (Ca^2+^) through N-methyl-D-aspartate (NMDA) receptors. Excessive Ca^2+^ can induce cell damage and neuronal death through various mechanisms. In summary, αKtx12 promotes cellular proliferation and/or neuroprotection (Figs. 5–6) in neurons through the aforementioned processes. This potential effect may contribute to functional recovery in cases of nervous system injury or disease.

αKtx12, whose biological effects were assessed by neuroproteomic analyses, proved to be a useful tool for characterizing neuroplasticity and neurotropism, according to the employed models, highlighting how blockade of the potassium channel can elicit more physiological responses than simple electrical impedance of the synapse.

## Conclusion

The intricate molecular landscape uncovered in this study is further shaped by the specific blockade of the Kv channel by the αKtx12 peptide, adding a layer of complexity to the observed neurobiological phenomena. The targeted action of the peptide on the Kv channel is a critical element in understanding the cascade of events leading to the metabolic shift and subsequent adaptations in neuronal function. This study highlighted the neuroprotective potential of the αKTx12 peptide, derived from the venom of the *Tityus bahiensis* scorpion, through the selective inhibition of the Kv1.3 potassium channel, however, as a limitation of this study, our focus on the Kv1.3 channel, although relevant, does not account for potential interactions of the peptide with other ion channels or molecular pathways that may also contribute to neuroprotection.

Through cellular viability, histological, and neuroproteomic analyses, we demonstrated that αKTx12 significantly promotes neuronal viability and the proliferation of pyramidal cells in the hippocampus. The multifaceted exploration of energy pathways, neurotrophic factors, and synaptic dynamics provides a comprehensive understanding of the intricate molecular landscape that governs neuronal function, the label-free proteomics approach, while informative, may miss low-abundance proteins or subtle molecular changes that could play crucial roles in the peptide’s neuroprotective mechanisms, and the short-term observation period in animal models might not reveal long-term effects of αKTx12, requiring further studies with extended timeframes. The implications of these findings extend to potential therapeutic avenues for neurodegenerative or neuroinflammation disorders, it is important to note that the peptide should be further studied in specific models of neuroinflammation and neurodegeneration to fully understand its therapeutic potential in these conditions; as the identified mechanisms provides insights into the adaptive strategies employed by neurons in response to perturbations. This study not only contributes to the expanding body of knowledge in neuroscience, but also opens new avenues for targeted interventions aimed at preserving and enhancing neuronal function in health and disease.

## Methodology

### Chromatographic fractionation

*Tityus bahiensis* (Tb) pooled lyophilized venom was provided by the Strategic Nucleus of Venoms and Antivenoms (NEVAS), Butantan Institute, São Paulo, Brazil. The first step was to fractionate the venom. Briefly, 3 mg of lyophilized Tb venom were resuspended in 0.1% trifluoroacetic acid (TFA) and centrifuged (10,000 x *g*) for 10 min, at 4° C. The supernatant was then analyzed and fractionated by Reversed-Phase High-Performance Liquid Chromatography (RP-HPLC) on a Shimadzu Prominence binary system (Shimadzu, Kyoto, Japan), coupled to a C4 analytical column (Supelco, 250 × 4.6 mm, 10 μm). UV detection was performed (SPDM 20 A, Shimadzu, λ = 214 nm) and separation was achieved by an optimized linear gradient of 0–100% solvent B (90% acetonitrile (ACN), containing 0.1% TFA) over A (0.1% TFA) for 60 min at a constant flow of 1 mL.min^− 1^.

A second chromatographic step of fraction F3 was necessary for further separation of the active molecules, now employing a C18 analytical column (Supelco, 250 × 4.6 mm, 5 μm), under a linear gradient of 0–60% solvent B (90% ACN, containing 0.1% TFA) over A (0.1% TFA) for 60 min at a constant flow of 1 mL.min^− 1^. UV detection was performed (SPDM 20 A, Shimadzu, λ = 214 nm).

### In-solution digestion

All samples subjected to proteomic analysis, including the tested peptide αKtx12 and hippocampal tissues, followed the same standardized digestion protocol to ensure consistency across experimental conditions. Fifty microliters of 50 mM ammonium bicarbonate were added to the lyophilized samples which were reduced by the addition of 2 µL, 100 mM of dithiotheitol (DTT), at 60° C for 30 min. The samples were then alkylated by adding 2 µL, 200 mM iodoacetamide, at room temperature for 30 min. The reaction was protected from light. Samples were digested by trypsin (1 µg, Trypsin Singles, Proteomics Grade, SIGMA-ALDRICH, St. Louis, MO, USA) overnight, at 37 °C. The reaction was stopped with 5 µL of acetic acid.

### Mass spectrometry and αKtx12 identification

The subfraction F3.4 was analyzed using a matrix-assisted laser desorption ionization-time of flight (MALDI-TOF) mass spectrometer (Axima Performance, Shimadzu, Kyoto, Japan). First, 1 µl of the subfraction was co-crystallized with 1 µl of sinapinic acid matrix (saturated solution prepared in 50% ACN/0.1% acetic acid) in the plate and dried at room temperature. The mass spectrum was obtained in the 50–20,000 mass/charge (m/z) range, in linear positive mode with laser power at 120.

Then in-solution digestion protocol was applied in the subfraction F3.4.

The sample then was analyzed by liquid chromatography-mass spectrometry in an ESI-IT-TOF instrument coupled to a UPLC 20 A Prominence (Shimadzu, Kyoto, Japan). Samples (15 µL aliquots) were loaded into a C18 column (Kinetex C18, 5 μm; 50 × 2.1 mm) and fractionated by a binary gradient employing as solvents (A) water: dimethyl sulfoxide (DMSO): water: acid (949: 50: 1) and (B) ACN: DMSO: water: acid (850: 50: 99: 1). An elution gradient of 0–40% B was applied for 35 min at a constant flow of 0.2 mL.min-1 after initial isocratic elution for 5 min. The eluates were monitored by a Shimadzu SPD-M20A PDA detector before being injected into the mass spectrometer.

The interface was kept at 4.5 kV and 275 °C. Detector operated at 1.95 kV and the argon collision induced fragmentation was set at 55 ‘energy’ value. MS spectra were acquired in positive mode, in the 350–1400 m/z range and MS/MS spectra were collected in the 50 to 1950 m/z range.

Raw LCD LCMSolution Shimadzu data were converted into MGF by the LCMSolution tool and then loaded into Peaks Studio V7.0 (BSI, Canada). Data were processed according to the following parameters: MS and MS/MS error mass were 0.1 Da; methionine oxidation and cysteine carbamidomethylation as variable and fixed modification, respectively; trypsin as cleaving enzyme; maximum missed cleavages (3), maximum variable PTMs per peptide (3) and non-specific cleavage (both); the false discovery rate was adjusted to ≤ 0.5%; only proteins with score ≥ 20 and containing at least 1 unique peptide were considered in this study. Data were analyzed against two protein databases “AnimalVenom” and “*Tityus bahiensis* transcriptomic” compiled on Jan/2024 and built by retrieving all UniProt entries associated with this taxon, although broad searches against the whole UniProt were performed, as well, as quality controls (data not shown). Built-in Peaks PTM and Spider algorithms were used to identify peptide mutations and/or isoforms.

### SH-SY5Y cell culture and viability

Tb venom, fractions and/or isolated peptides (subfractions) were tested under the following conditions: 5 × 10^4^ SH-SY5Y cells/well (ECACC, Sigma Aldrich, St. Louis, MO, USA) were maintained in a humidified 5% CO_2_ incubator at 37 °C. Cells were incubated with 10–1 µg Tb venom or fractions F1-5, or with 0.5 µg of subfractions F3.1-3.5. Additionally, positive damage (DMSO) and negative (untreated) controls were performed. After 48 h of treatment, the cell viability was determined by the 3-(4,5-dimethylthiazol-2-yl)-2,5-diphenyltetrazolium bromide (MTT) assay, in which the medium was discarded, and the reagent was incubated for 4 hours at a concentration of 0.5 mg.mL^− 1^. The blue formazan product was dissolved in DMSO, and quantification of formazan was performed (λ = 540 nm) using an automatic microplate reader (SLT, Austria). Results are presented as % viable cells, versus fraction/peptide concentration.

### Stereotaxic surgery

For the present study, the inclusion criteria were established to ensure the homogeneity and health of the animals used, thus guaranteeing the validity of the experimental results. The animals included in the study were male Wistar rats, sourced from the Central Animal Facility of the Butantan Institute and housed in the Laboratory of Pharmacology for the in vivo assays. They had to be between 8 and 12 weeks old and weigh between 260 and 280 g. Only healthy animals, without signs of disease or infection, were selected, with a prior evaluation to ensure the absence of behavioral or physical anomalies. The animals were housed under standardized conditions, with a 12-hour light/dark cycle, a controlled temperature of 22 ± 2 °C, and ad libitum access to water and food. Additionally, only animals that had not been subjected to any other experimental procedures previously were considered for inclusion in the study. The exclusion criteria were defined to eliminate variables that could compromise the integrity of the study results. Animals showing signs of disease, infection, or physical anomalies detected during the initial screening were excluded. Animals that did not fit within the specified weight range (260–280 g) or age range (8–12 weeks) were also excluded. Those exhibiting aggressive or anomalous behavior that could interfere with the experimental procedures were not included. The sample size used in this study was established based on protocols agreed upon with the Animal Ethics Committee (CEUA). A number of four or six animals per experimental group (*n* = 4 or 6) was chosen to ensure the reproducibility and statistical validity of the results. All experimental procedures and methods were approved and in accordance with the ethical principles in animal research adopted by the Brazilian Society of Animal Science and the National Brazilian Legislation No. 11.794/08. Animal care experimental procedures were previously approved by the Institutional Ethics Committee for Experimental Animals (No. 3637060619) on July 28, 2019. To ensure the impartiality and validity of the results, researchers responsible for administering treatments and evaluating outcomes were blinded to the group allocations. This blinding was maintained throughout all experimental stages, including data collection and analysis. In this way, we ensured that observations and interpretations were conducted objectively, without the influence of prior knowledge of the assigned treatments.

The animals were anesthetized with a mixture of 10% ketamine and 2% xylazine (1.3 mg.kg^− 1^, i.p.) and fixed in a stereotaxic apparatus. Stainless steel guide cannulas were chronically implanted into one side of the dorsal hippocampus, coordinates AP -4.8, L -3.2, V -2.5, according to Paxinos and Watson Atlas^48^.The system was anchored to the skull with jeweler screws and dental acrylate. After surgery, the animals were housed individually and allowed to recover for a period of 4–5 days. Subsequently, αKtx12 (2 µg, suspended in 2 µL of Ringer’s solution), Ringer’s solution (2 µL) or LPS (10 µg/µL) were slowly injected (0.25 µL.s^− 1^) in the rat hippocampus using a 30-G needle connected to a 5 µL Hamilton syringe. The animals were used for histologic (*n* = 6/group) and neuroproteomic (*n* = 4/group) analysis.

### Surgical removal of brain structures and sample preparation for neuroproteomic analysis

The proteomic analyses of the cerebral tissues were performed in animals treated with αKtx12 (experimental group, *n* = 4), Ringer’s solution (negative control, *n* = 4), and lipopolysaccharide (LPS, positive inflammation control, *n* = 4). Animals were euthanized 6 h after treatment and the cerebral structures cortex, hippocampus and cerebellum were removed, frozen and stored in 0.1% TFA. This time point was chosen because previous studies from our laboratory show that most inflammatory cytokines are altered between 4 and 6 h after the application of scorpion venom or toxins (Beraldo Neto, 2018; Rodriguez et al., 2015, Dorce et al., 2015). At the time of analysis, the samples were homogenized by a sonication and centrifuged (14,170 × g, 4 °C), for 15 min. 300ug (total protein, determined by nanodrop with 280 nm read) of supernatant from each group was used for analyses.

### Surgical removal of hippocampus for histological analysis

The histological analyses of hippocampus were performed in animals treated with αKtx12 (experimental group) or Ringer’s solution (negative control). The animals were anesthetized with carbon dioxide (CO_2_) one week after treatment (this is the time required to verify whether neurodegeneration occurs) and perfused with phosphate-buffered saline (PBS) and 10% formalin solution via cardiac puncture. After decapitation, brains were removed and stored in formalin for at least 1 week, then processed (successive alcohol baths ending with xylol overnight) and embedded in Paraplast (Oxford Labware, St. Louis, MO, USA). Coronal brain sections of 10 μm were cut from a 700 μm brain block containing the cannula track. The slices were mounted on a glass slide and stained with cresyl violet. Five slices were counted per animal. The number of pyramidal cells in the CA1, CA3 and CA4 hippocampal areas was analyzed by light microscopy using a 40X magnification objective. A two-dimensional cell count was manually performed using a 100 × 100 μm reticulum. Only pyramidal neurons localized inside the reticulum area, with a visible nuclei and nucleolus were considered intact.

### Neuroproteomics

In-solution digestion was performed on the hippocampus samples. Samples were cleaned up using C18 ZipTips (Merck KGaA, Darmstadt, Germany). One microliter of the resulting solution, containing the tryptic peptides, was subjected to nano-ESI-LC-MS/MS using a Dionex Ultimate 3000 RSLCnano (Thermo Fisher Scientific, Waltham, MA, USA) coupled to an Impact II Mass spectrometer (Bruker Daltonics, Bremen, Germany). The samples were injected in a nano-trap Acclaim PepMap (Dionex-C18, 100 Å, 75 μm × 2 cm) in 2% solvent A2 (0.1% formic acid) for 2 min, under a 5 µL·min^− 1^ flow rate. Elution was performed by a linear gradient of 5–40% of solvent B2 (0.1% formic acid in ACN), for 120 min, under 350 nL min^− 1^ flow rate. Mass spectra were acquired in positive mode. MS and MS/MS scans were acquired at 2 Hz, in a 50–2000 m/z range. CID energy ramped between 7 and 70 eV. Data were processed by Peaks Studio 8.5 (Bioinformatics Solution Inc., Waterloo, ON, Canada), and searched against the UniProt *Rattus norvergicus* Reference Proteome (UP000002494).

Label-free analyses were performed using the PEAKS Q module in PEAKS Studio 8.5, which first aligned the acquired LC-MS/MS chromatographic data and integrated peptide identification database searches with Peaks DB for peptide quantification. Changes in relative abundance levels were calculated for each identified peptide by comparing peak areas calculated from the extracted ion chromatograms (TIC). The following settings were used: precursor ion width adjusted to 15 ppm; normalization of the area under the curve (AUC) for each identified peptide based on the total ion count (TIC); estimation of the peptide ratio between groups filtered by peptides automatically detected.

The identified proteins were evaluated in relation to the quantity of peptides available for detection, and ranked according to their tissular availability; this was directed to evaluate possible alterations in the metabolic pathways^49,50^.

### Statistical analysis

The cell viability assays (MTT) were analyzed using one-way ANOVA followed by Dunnett’s post-hoc test for the initial set of comparisons. For the second analysis, when the peptide was isolated, a t-test was used, all statistical analyses were conducted using GraphPad software, with statistical significance set at *p* < 0.05 to detect meaningful differences among the venom fractions. Histological evaluations employed the unpaired t-test through the same software, applying a significance threshold of *p* < 0.05 to compare pyramidal neuron counts between control and treatment groups. Additionally, a variance test was performed within the groups to ensure homogeneity and normalization of the data. Proteomic analyses were conducted using the PEAKS software, relying on its built-in statistical algorithms to identify proteins differentially expressed across experimental conditions.

## Electronic supplementary material

Below is the link to the electronic supplementary material.

Supplementary Material 1

## Data Availability

Raw data of mass spectrometry analysis is available at https://repository.jpostdb.org/entry/JPST002939.0.
