# Supplementary material for: Unraveling neuroprotection with Kv1.3 potassium channel blockade by a scorpion venom peptide
Source: Sci Rep. 2024 Nov 13;14:27888. doi: 10.1038/s41598-024-79152-1 (PMC11561340; doi:10.1038/s41598-024-79152-1)
Supplement: Supplementary file 1 — Supplementary Material 1 [file 41598_2024_79152_MOESM1_ESM.docx]

**Supplementary Figures**

A


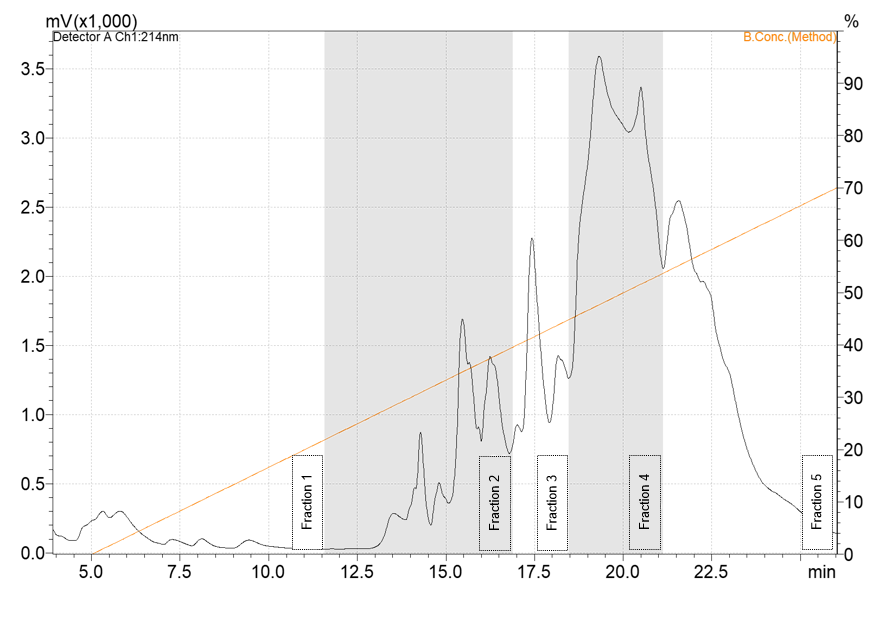
B


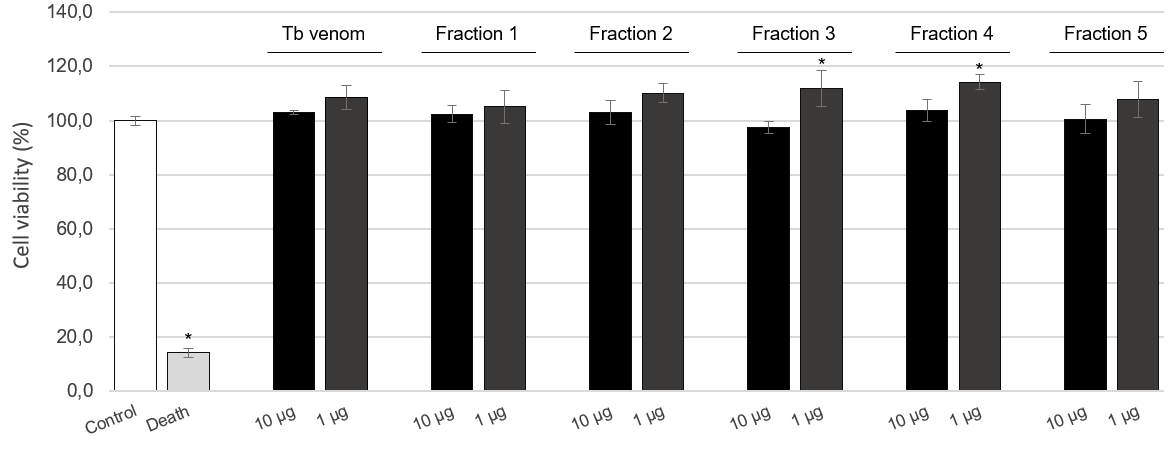


**Supplementary Figure S1**. (A) Representative cromatographic profile of *T. bahiensis* venom in C4-RP-HPLC. (B) Cell viability assay by MTT method in SH-SY5Y cell line. Total *T. bahiensis* venom and corresponding fractions obtained by C4-RP-HPLC. ANOVA followed by Dunnett's test *statistical difference in relation to the control p<0.05.

A
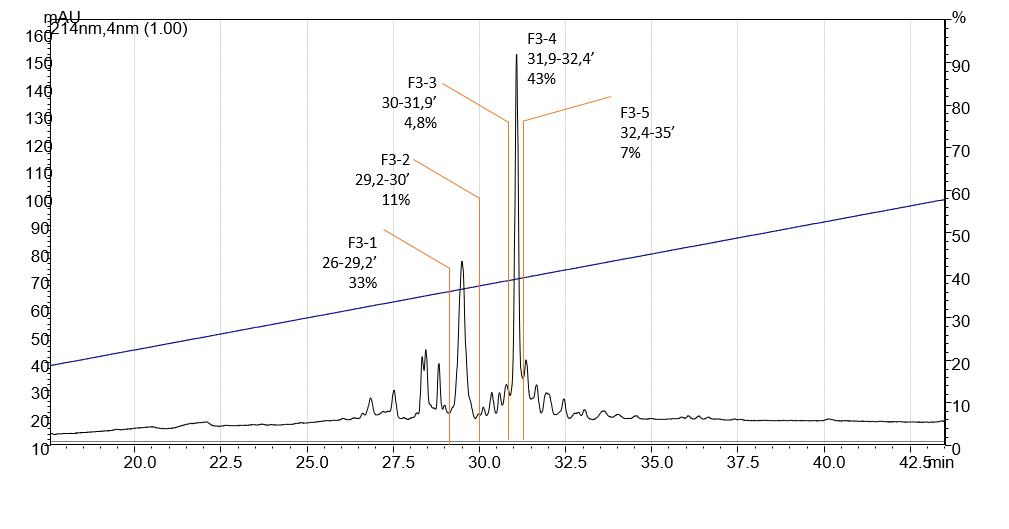
B
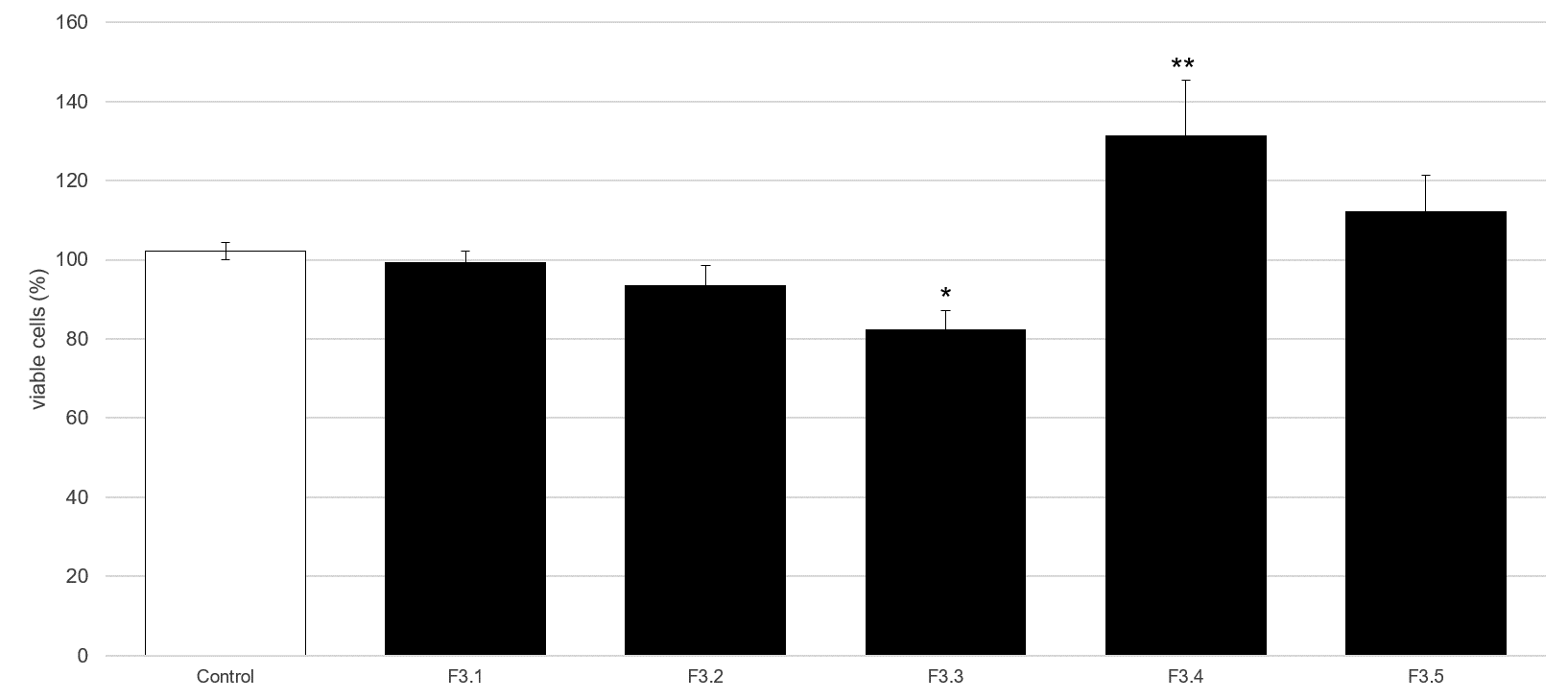


**Supplementary Figure S2**. (A) Representative C18-RP-HPLC profile of fraction 3 from *T. bahiensis* venom, identification of subfractions, retention time, and fraction yield in percentage. (B) Cell viability assay using the MTT Method in SH-SY5Y Cell Line of 0.5 µg of each subfractions obtained from fraction 3 of *T. bahiensis* venom by C18-RP-HPLC technique. The t-test was conducted for each fraction in comparison to the control * p<0.05 and ** p<0.01.


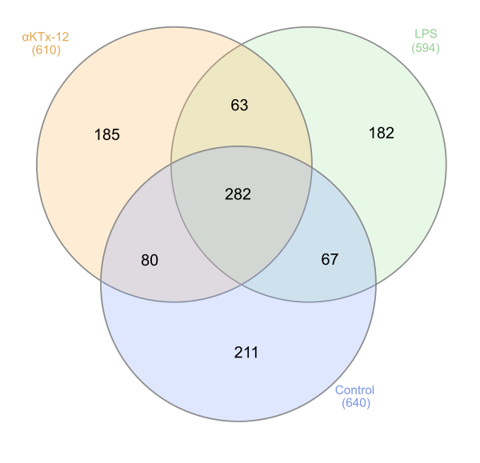


**Supplementary Figure S3.** Venn diagram indicating the quantities of unique and shared proteins found in each group analyzed by differential proteomics.
